# Identification of biomarkers and pathways for the SARS-CoV-2 infections in obstructive sleep apnea patients based on machine learning and proteomic analysis

**DOI:** 10.1186/s12890-024-02921-1

**Published:** 2024-03-05

**Authors:** Hong Luo, Jisong Yan, Rui Gong, Dingyu Zhang, Xia Zhou, Xianguang Wang

**Affiliations:** 1 Department of Tuberculosis and Respiratory, Wuhan Jinyintan Hospital, Tongji Medical College, Huazhong University of Science and Technology, Wuhan, China; 2 Hubei Clinical Research Center for Infectious Diseases, Wuhan, China; 3 Wuhan Research Center for Communicable Disease Diagnosis and Treatment, Chinese Academy of Medical Sciences, Wuhan, China; 4 Joint Laboratory of Infectious Diseases and Health, Wuhan Institute of Virology and Wuhan Jinyintan Hospital, Chinese Academy of Sciences, Wuhan, China; 5 Division of Life Sciences and Medicine, The First Affiliated Hospital of USTC, University of Science and Technology of China (USTC), Hefei, Anhui China; 6 Center for Translational Medicine, Wuhan Jinyintan Hospital, Tongji Medical College, Huazhong University of Science and Technology (HUST), Wuhan, Hubei China; 7 Department of Critical Care Medicine, Union Hospital, Tongji Medical College, Huazhong University of Science and Technology (HUST), Wuhan, Hubei China

**Keywords:** Obstructive sleep apnea, COVID-19, Feature genes, Hub genes, Pathogenesis

## Abstract

**Background:**

The prevalence of obstructive sleep apnea (OSA) was found to be higher in individuals following COVID-19 infection. However, the intricate mechanisms that underscore this concomitance remain partially elucidated. The aim of this study was to delve deeper into the molecular mechanisms that underpin this comorbidity.

**Methods:**

We acquired gene expression profiles for COVID-19 (GSE157103) and OSA (GSE75097) from the Gene Expression Omnibus (GEO) database. Upon identifying shared feature genes between OSA and COVID-19 utilizing LASSO, Random forest and Support vector machines algorithms, we advanced to functional annotation, analysis of protein–protein interaction networks, module construction, and identification of pivotal genes. Furthermore, we established regulatory networks encompassing transcription factor (TF)-gene and TF-miRNA interactions, and searched for promising drug targets. Subsequently, the expression levels of pivotal genes were validated through proteomics data from COVID-19 cases.

**Results:**

Fourteen feature genes shared between OSA and COVID-19 were selected for further investigation. Through functional annotation, it was indicated that metabolic pathways play a role in the pathogenesis of both disorders. Subsequently, employing the cytoHubba plugin, ten hub genes were recognized, namely TP53, CCND1, MDM2, RB1, HIF1A, EP300, STAT3, CDK2, HSP90AA1, and PPARG. The finding of proteomics unveiled a substantial augmentation in the expression level of HSP90AA1 in COVID-19 patient samples, especially in severe conditions.

**Conclusions:**

Our investigation illuminate a mutual pathogenic mechanism that underlies both OSA and COVID-19, which may provide novel perspectives for future investigations into the underlying mechanisms.

**Supplementary Information:**

The online version contains supplementary material available at 10.1186/s12890-024-02921-1.

## Introduction

During the past three years, an extensive number of individuals have faced the challenging period of coronavirus disease outbreaks. The latest estimates from the World Health Organization indicate a staggering 765,222,932 confirmed cases of COVID-19 as of May 3rd, 2023, with 6,921,614 reported fatalities (https://covid19.who.int/). This has placed a significant burden on healthcare systems worldwide. Severe acute respiratory syndrome coronavirus 2 (SARS-CoV-2), the causative agent of COVID-19, primarily affects the upper respiratory tract and lung tissues. Previous studies have identified older age, diabetes, severe asthma, hypertension, obesity, and cardiovascular and cerebrovascular comorbidities as high-risk factors associated with COVID-19 infection and unfavorable prognosis [1–3]. Intriguingly, several of these comorbidities, such as hypertension, diabetes mellitus, obesity, and vascular disease, have been shown a strong association with OSA [4–6]. Hence, exploring shared pathogenic mechanisms between these two diseases has captured significant interest among researchers and clinicians.

OSA is a disorder characterized by recurring episodes of pharyngeal airway collapse, leading to intermittent partial or complete cessation of breathing during sleep. This condition has been strongly associated with various cardiovascular, cerebrovascular, and metabolic disorders. In recent years, extensive clinical observational studies have indicated that individuals with OSA may be more susceptible to COVID-19 infection and experience poorer outcomes [7–15]. Furthermore, two meta-analyses have revealed that OSA is associated with a 2.0 and 1.7 times increased risk of severe COVID-19, respectively [16, 17].

Nevertheless, the precise pathological mechanisms underlying the association between OSA and the susceptibility to COVID-19 infection as well as the development of COVID-19 complications remain inadequately elucidated. Several investigations propose an intricate interplay involving intermittent hypoxia, oxidative stress, sympathetic activation, inflammation, endothelial dysfunction, and associated complications [17, 18]. To delve further into these intricate mechanisms, a case–control study has unveiled a correlation between sleep-related hypoxemia and an augmented likelihood of adverse COVID-19 outcomes. This discovery implies a potential linkage between COVID-19-induced hypoxia and pre-existing sleep-related hypoxemia. Furthermore, given the concurrent involvement and impact on the respiratory system in both conditions, certain studies postulate that individuals with pre-existing OSA are at an elevated risk of morbidity and mortality upon exposure to SARS-CoV-2 due to an excessive inflammatory response [19, 20]. Moreover, a review conducted by Mashaqi et al. suggested that alterations in the gut microbiota, which occur in the context of COVID-19 and OSA, can exacerbate systemic inflammation by facilitating the translocation of microorganisms across a compromised intestinal barrier [8]. Overall, the current theories and hypotheses fail to provide a comprehensive elucidation of the intrinsic molecular mechanisms underlying the association between OSA and COVID-19. Comprehending the potential interconnections and plausible molecular pathways connecting these two conditions is imperative for the effective management of COVID-19 patients with concomitant OSA and for the identification of viable therapeutic interventions.

In recent times, the clinical utilization of bioinformatics has witnessed a surge, enabling the exploration of intricate disease mechanisms. Its remarkable advantages lie in its capacity to prognosticate the structure, prognosis, functionality, and evolution of uncharted genes and proteins, while also facilitating the screening of medications and vaccines from extensive repositories of sequence data. Consequently, the objectives of this investigation encompassed an examination of the plausible association between COVID-19 and OSA, along with an in-depth exploration of the molecular mechanisms that underpin this comorbidity, employing a bioinformatics framework.

## Materials and methods

### Datasets information

The dataset denoted as GSE157103 encompasses a COVID-19 cohort, encompassing gene expression profiles and accompanying clinical data obtained from a cohort comprising 100 COVID-19 patients, alongside 26 non-COVID-19 patients [21]. Additionally, GSE75097 has captured gene expression profiles from 28 individuals afflicted with OSA, in conjunction with 6 healthy controls [22].

### Screening feature genes based on LASSO, random forest and support vector machine algorithms

In COVID-19, we conducted gene screening employing the LASSO, random forest, and support vector machine algorithms. The resultant intersection set was derived from these methodologies to ascertain the distinctive feature genes. Furthermore, similar gene screening procedures were applied to the realm of OSA utilizing the LASSO, random forest, and support vector machine algorithms. Following this, the R package "venn" was employed to discern the overlapping feature genes that are shared between the datasets of COVID-19 and OSA.

### Functional annotation of feature genes

Enrichr is an internet-based application designed for conducting gene set enrichment analysis, enabling users to investigate gene lists derived from high-throughput experiments and gain a comprehensive understanding of the biological implications and underlying mechanisms. This versatile tool incorporates over 100 gene set libraries, encompassing diverse categories such as biological pathways, Gene Ontology, transcription factor targets, protein domains, and more [23]. In this study, we employed the Enrichr tool to carry out functional annotation of the shared feature genes, encompassing Gene Ontology (GO) analysis and KEGG pathway analysis. Statistical significance was defined as a *p*-value less than 0.05.

### Network construction for protein–protein interactions, hub gene identification and module analysis

In this investigation, we utilized the STRING online platform to investigate the interconnectedness among the shared feature genes [24]. Furthermore, the Cytoscape software was employed to illustrate and visually represent the interaction relationships among these genes [25]. To identify core nodes and modules within the network, we utilized the CytoHubba plugin, which also facilitated the identification of hub genes [26]. More specifically, the MCC algorithm of the CytoHubba plugin was employed to ascertain the hub genes, while the MCODE plugin for Cytoscape aided in the identification of core functional modules within the protein–protein interaction network [27].

### Identification of TF-gene interactions and construction of TF-miRNA interaction network

To unravel the complex interplay between transcription factors (TFs), genes, and miRNAs, we leveraged the extensive resources provided by the ENCODE database and RegNetwork database, which were accessible through the NetworkAnalyst platform. This enabled us to discover TF-gene interactions and construct a TF-miRNA interaction network [28, 29].

### Screening potential drugs

By utilizing the DSigDB database, we predicted plausible potential drugs that may be associated with the hub genes. DSigDB serves as a comprehensive repository of drug-gene expression profile data sourced from public databases. It provides researchers with data analysis tools and visualization interfaces to explore the relationship between drugs and gene expression, facilitating the identification of promising therapeutic targets and drugs [30]. Leveraging this database, we predicted potential target drugs linked to the hub genes, holding promise for the treatment of OSA and COVID-19.

### Validation of hub genes based on proteomics and immune infiltration analysis

Drawing upon the findings elucidated in our previously disseminated proteomic investigations, conducted under the aegis of our research team, we embarked upon the validation of the expression gradients exhibited by the corresponding proteins linked with the hub genes within both the COVID-19 tissue samples and serum samples [31]. Subsequently, we engaged the CIBERSORT algorithm to quantitatively measure the extent of infiltration demonstrated by 22 distinct immune cell populations across the panorama of COVID-19 samples and OSA samples [32]. To further delineate the intricate interplay between the central genes and the immunological attributes, a comprehensive correlation analysis was conducted. To discern the diagnostic efficacy of the central genes in both COVID-19 and OSA, Receiver Operating Characteristic (ROC) curves were meticulously constructed and the Area Under the Curve (AUC) was computed individually for evaluation purposes.

## Results

### Identifying feature genes

**Fig. 1:**
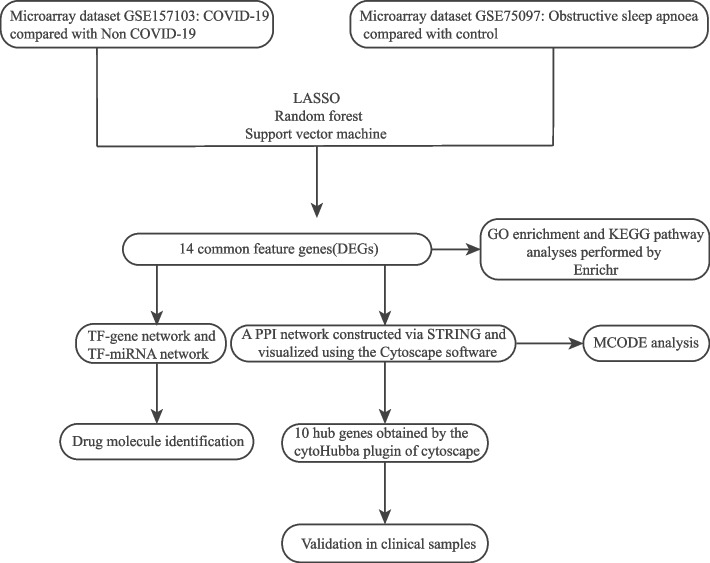
Workflow diagram of this study

The methodology employed in this investigation is delineated in Fig. 1, elucidating the process of discerning feature genes within the GSE157103 and GSE75097 datasets. A collective sum of 25 feature genes and 24 feature genes were respectively identified within these datasets (Figure S1; Figure S2). Through the implementation of intersection analysis, a set of fourteen genes commonly differentially expressed was obtained.

### Functional enrichment analysis

**Fig. 2:**
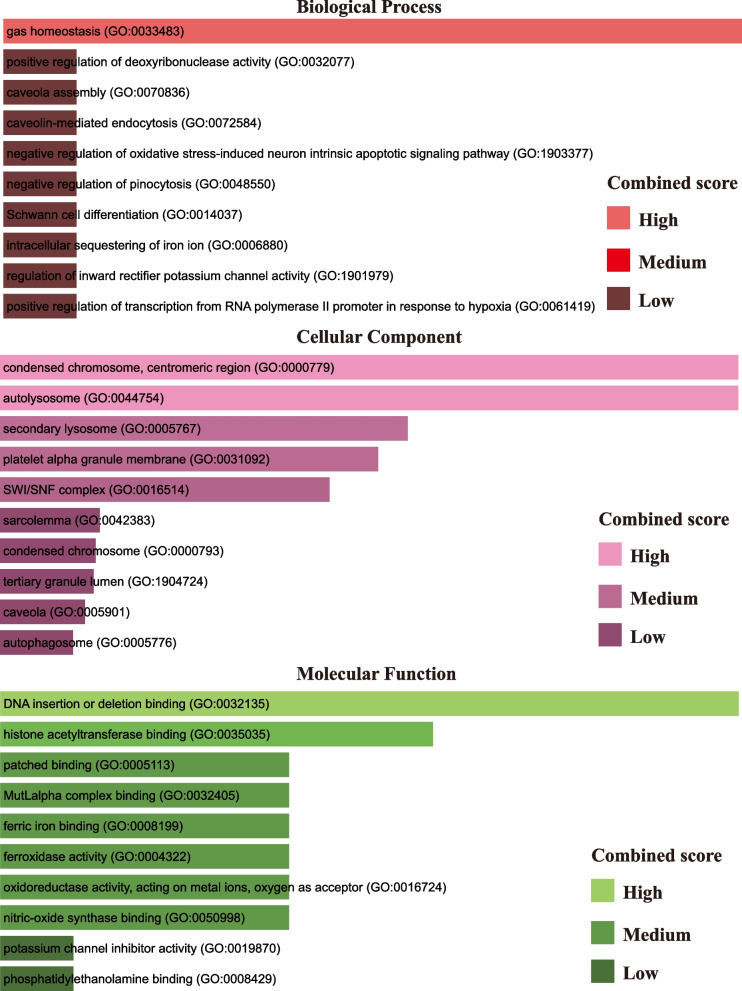
Biological process, molecular function and cellular component related GO terms identification result according to combined score. The higher the enrichment score, the higher number of genes are involved in a certain ontology

In this study, we conducted an enrichment analysis utilizing the web-based tool Enrichr, which facilitated the identification of multiple enriched GO categories. The outcomes of the enrichment analysis revealed numerous significant biological processes, cellular components, and molecular functions that potentially hold key roles in the studied system. Noteworthy enriched biological processes encompassed gas homeostasis, caveola assembly, and Schwann cell differentiation, suggesting their relevance in the system. Moreover, various molecular functions such as ferric iron binding, oxidoreductase activity, and phosphatidylethanolamine binding were enriched, highlighting their potential significance in the system. Furthermore, the analysis yielded several significant cellular components, including the condensed chromosome, centromeric region, and autolysosome, implicating their involvement in the studied system. Notably, the enrichment analysis also highlighted the significance of the SWI/SNF complex and caveola. (Fig. 2; Table 1).

**Table 1:** GO terms, GO pathways and their corresponding *P*-values and genes for common differentially expressed genes

Category	GO ID	GO pathways	*P*-value	Genes
GO Biological Process	GO:0033483	gas homeostasis	9.54E-06	CAV1;HIF1A
	GO:0032077	positive regulation of deoxyribonuclease activity	0.003495399	PCNA
	GO:0070836	caveola assembly	0.003495399	CAV1
	GO:0072584	caveolin-mediated endocytosis	0.003495399	CAV1
	GO:1,903,377	negative regulation of oxidative stress-induced neuron intrinsic apoptotic signaling pathway	0.003495399	HIF1A
	GO:0048550	negative regulation of pinocytosis	0.003495399	CAV1
	GO:0014037	Schwann cell differentiation	0.003495399	NF1
	GO:0006880	intracellular sequestering of iron ion	0.003495399	FTH1
	GO:1,901,979	regulation of inward rectifier potassium channel activity	0.003495399	CAV1
	GO:0061419	positive regulation of transcription from RNA polymerase II promoter in response to hypoxia	0.003495399	HIF1A
GO Cellular Component	GO:0000779	condensed chromosome, centromeric region	0.006979474	NCAPD2
	GO:0044754	autolysosome	0.006979474	FTH1
	GO:0005767	secondary lysosome	0.01114544	FTH1
	GO:0031092	platelet alpha granule membrane	0.011838189	CD109
	GO:0016514	SWI/SNF complex	0.013222334	RB1
	GO:0042383	sarcolemma	0.03580243	CAV1
	GO:0000793	condensed chromosome	0.03715538	NCAPD2
	GO:1,904,724	tertiary granule lumen	0.037831194	FTH1
	GO:0005901	caveola	0.041203662	CAV1
	GO:0005776	autophagosome	0.047246461	FTH1
GO Molecular Function	GO:0032135	DNA insertion or deletion binding	0.003495399	PCNA
	GO:0035035	histone acetyltransferase binding	9.48E-05	PCNA;HIF1A
	GO:0005113	patched binding	0.004890388	CAV1
	GO:0032405	MutLalpha complex binding	0.004890388	PCNA
	GO:0008199	ferric iron binding	0.004890388	FTH1
	GO:0004322	ferroxidase activity	0.004890388	FTH1
	GO:0016724	oxidoreductase activity, acting on metal ions, oxygen as acceptor	0.004890388	FTH1
	GO:0050998	nitric-oxide synthase binding	0.004890388	CAV1
	GO:0019870	potassium channel inhibitor activity	0.006283564	CAV1
	GO:0008429	phosphatidylethanolamine binding	0.006283564	NF1

**Fig. 3:**
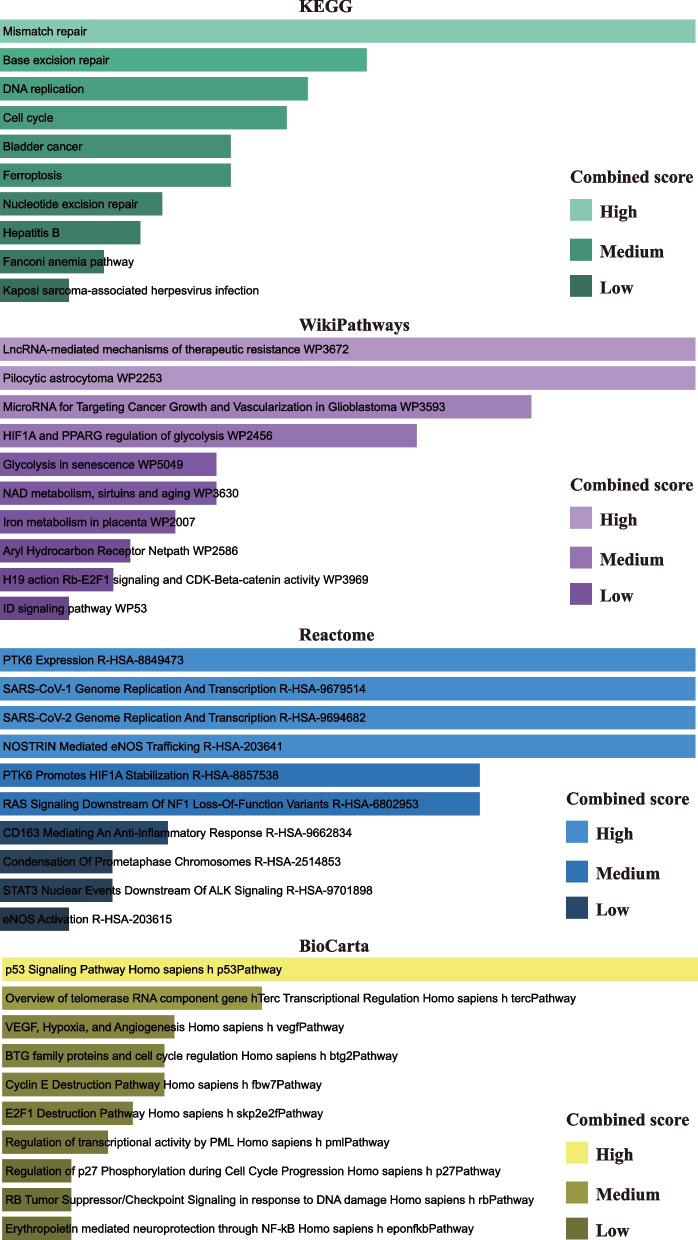
Pathway analysis result identification through KEGG, WikiPathways, Reactome and BioCarta. The results of the pathway terms were identified through the combined score

The enrichment analysis revealed noteworthy pathways derived from four comprehensive databases: KEGG, WikiPathways, Reactome, and BioCarta. Among these databases, the prominent pathways identified encompassed DNA repair and replication pathways, regulation of the cell cycle, as well as pathways associated with cancer, such as bladder cancer, Fanconi anemia pathway, and Kaposi sarcoma-associated herpesvirus infection. Additionally, several pathways pertaining to metabolism, aging, and gene regulation were also identified. Collectively, these findings provide valuable insights into the intricate molecular mechanisms that underlie diverse biological processes (Fig. 3; Table 2).

**Table 2:** Top pathways from KEGG, WikiPathways, Reactome and BioCarta databases and their corresponding *P*-values and genes for common differentially expressed genes

Databases	Pathways	*P*-value	Genes
KEGG	Mismatch repair	0.015985227	PCNA
	Base excision repair	0.022861069	PCNA
	DNA replication	0.024915106	PCNA
	Cell cycle	0.003304756	RB1;PCNA
	Bladder cancer	0.028329595	RB1
	Ferroptosis	0.028329595	FTH1
	Nucleotide excision repair	0.032412327	PCNA
	Hepatitis B	0.005566263	RB1;PCNA
	Fanconi anemia pathway	0.03715538	UBE2T
	Kaposi sarcoma-associated herpesvirus infection	0.007811021	RB1;HIF1A
WikiPathways	LncRNA-mediated mechanisms of therapeutic resistance WP3672	0.00419312	HIF1A
	Pilocytic astrocytoma WP2253	0.00419312	NF1
	MicroRNA for Targeting Cancer Growth and Vascularization in Glioblastoma WP3593	0.004890388	HIF1A
	HIF1A and PPARG regulation of glycolysis WP2456	0.005587202	HIF1A
	Glycolysis in senescence WP5049	0.007674931	RB1
	NAD metabolism, sirtuins and aging WP3630	0.007674931	HIF1A
	Iron metabolism in placenta WP2007	0.008369936	FTH1
	Aryl Hydrocarbon Receptor Netpath WP2586	0.000462719	RB1;NF1
	H19 action Rb-E2F1 signaling and CDK-Beta-catenin activity WP3969	0.009758591	RB1
	ID signaling pathway WP53	0.01114544	RB1
Reactome	PTK6 Expression R-HSA-8849473	0.003495399	HIF1A
	SARS-CoV-1 Genome Replication And Transcription R-HSA-9679514	0.003495399	RB1
	SARS-CoV-2 Genome Replication And Transcription R-HSA-9694682	0.003495399	RB1
	NOSTRIN Mediated eNOS Trafficking R-HSA-203641	0.003495399	CAV1
	PTK6 Promotes HIF1A Stabilization R-HSA-8857538	0.00419312	HIF1A
	RAS Signaling Downstream Of NF1 Loss-Of-Function Variants R-HSA-6802953	0.00419312	NF1
	CD163 Mediating An Anti-Inflammatory Response R-HSA-9662834	0.006283564	PLK2
	Condensation Of Prometaphase Chromosomes R-HSA-2514853	0.006979474	NCAPD2
	STAT3 Nuclear Events Downstream Of ALK Signaling R-HSA-9701898	0.006979474	HIF1A
	eNOS Activation R-HSA-203615	0.007674931	CAV1
BioCarta	p53 Signaling Pathway Homo sapiens h p53Pathway	3.53E-05	RB1;PCNA
	Overview of telomerase RNA component gene hTerc Transcriptional Regulation Homo sapiens h tercPathway	0.004890388	RB1
	VEGF, Hypoxia, and Angiogenesis Homo sapiens h vegfPathway	0.000195723	CAV1;HIF1A
	BTG family proteins and cell cycle regulation Homo sapiens h btg2Pathway	0.006283564	RB1
	Cyclin E Destruction Pathway Homo sapiens h fbw7Pathway	0.006283564	RB1
	E2F1 Destruction Pathway Homo sapiens h skp2e2fPathway	0.006979474	RB1
	Regulation of transcriptional activity by PML Homo sapiens h pmlPathway	0.007674931	RB1
	Regulation of p27 Phosphorylation during Cell Cycle Progression Homo sapiens h p27Pathway	0.009064489	RB1
	RB Tumor Suppressor/Checkpoint Signaling in response to DNA damage Homo sapiens h rbPathway	0.009064489	RB1
	Erythropoietin mediated neuroprotection through NF-kB Homo sapiens h eponfkbPathway	0.009064489	HIF1A

### Protein–protein interaction network

**Fig. 4:**
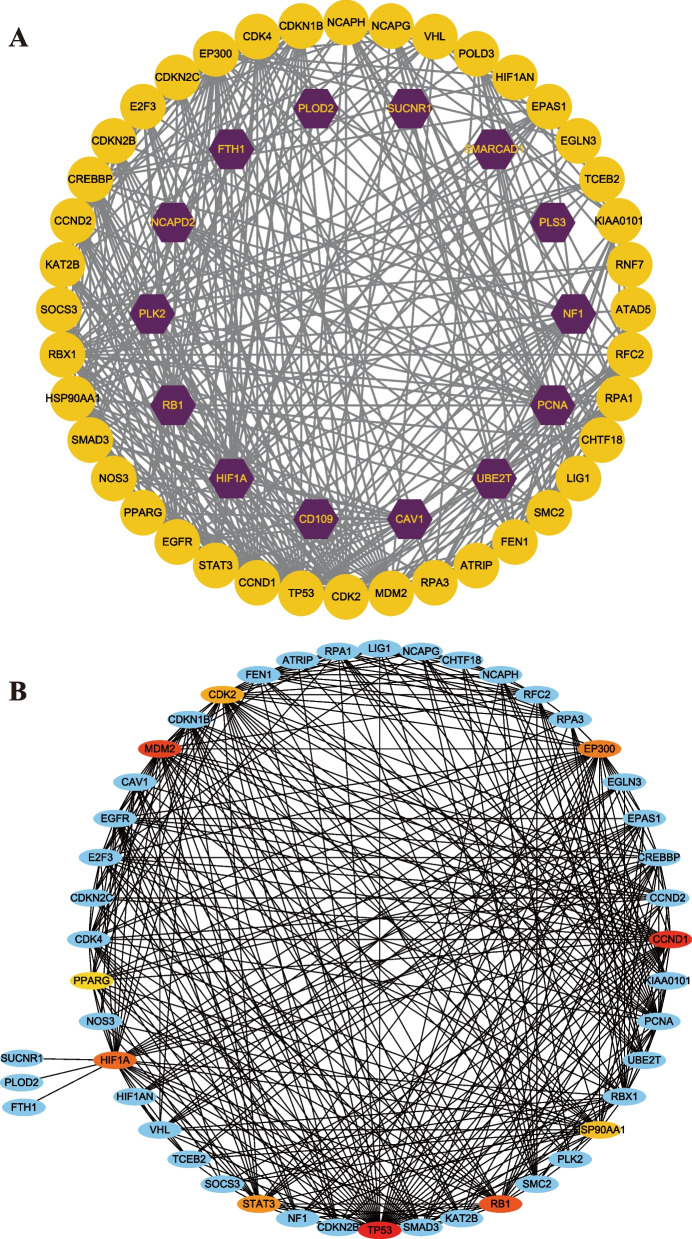
PPI network diagram and selection of hub genes. **A** Protein–protein interactions (PPIs) network for identified common feature genes that are shared by two diseases (COVID-19 and OSA). Purple nodes indicate genes with the top 10 interaction degree in the network. Nodes in yellow color indicate common feature genes and edges specify the interconnection in the middle of two genes. **B** Detection of hub genes from the PPIs network of common feature genes. A redder color means a higher degree of interaction

**Fig. 5:**
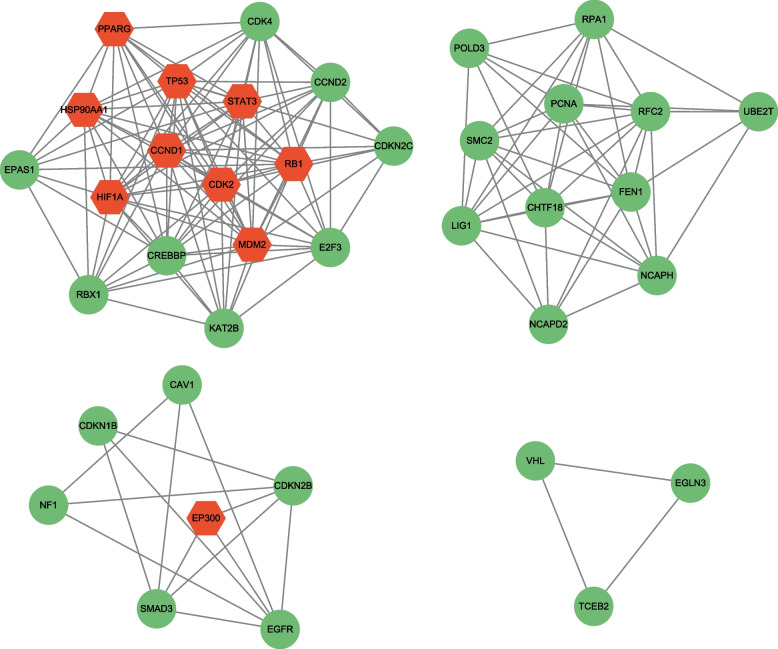
Identification of four significant gene modules based on MCODE plugin. Nodes in yellow color indicate hub genes

The protein–protein interaction network visually illustrates the tightly interconnected relationships among the common feature genes (Fig. 4A). Utilizing the cytoHubba plugin, we identified the top 10 central genes within the network, namely TP53, CCND1, MDM2, RB1, HIF1A, EP300, STAT3, CDK2, HSP90AA1, and PPARG (Fig. 4B). Furthermore, employing the MCODE plugin, we extracted four significant gene modules (Fig. 5). Notably, Module 1 encompasses all of the hub genes, with the exception of EP300.

### TF-gene interactions and construction of TF-miRNA interaction network

**Fig. 6:**
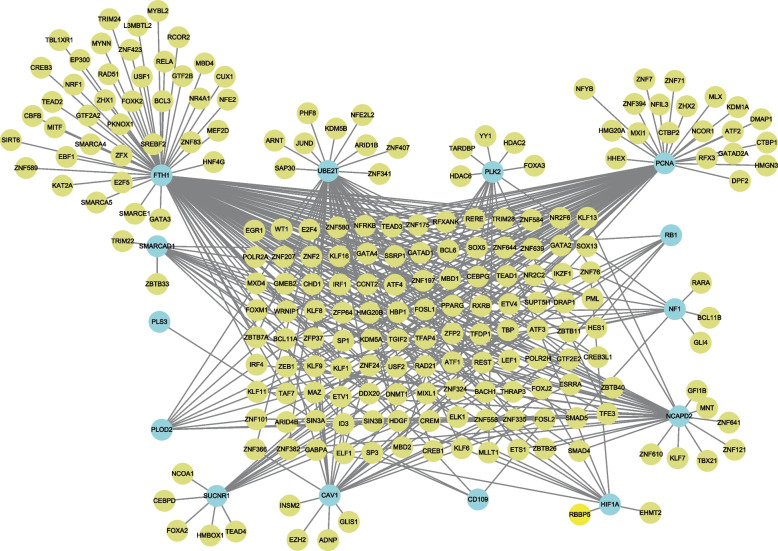
Network for TF-gene interaction with hub genes. The highlighted blue color node represents the hub genes and other nodes represent TF-genes

**Fig. 7:**
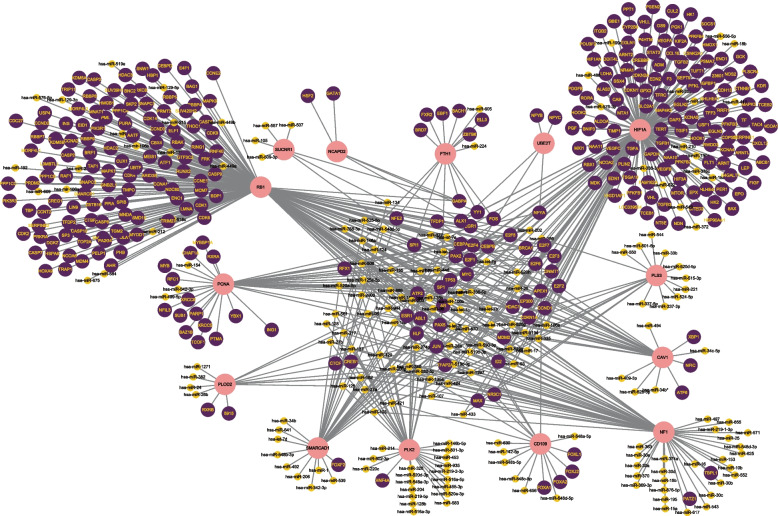
The network presents the TF-miRNA coregulatory network. The nodes in pink color are the hub genes, a yellow node represents miRNAs and other nodes indicate TF-genes

The NetworkAnalyst tool was employed to predict TFs capable of interacting with the fourteen commonly expressed feature genes, and to construct a visually informative network diagram depicting these interaction relationships. The resultant network comprised 213 TFs, 227 nodes, and 402 edges. Notably, FTH1 and PCNA emerged as potential key players in the context of COVID-19 infection complicated by OSA (Fig. 6). Moreover, a TF-miRNA co-regulation network was constructed, which facilitated the prediction of connections among miRNAs, TFs, and hub genes. This comprehensive network encompassed 529 nodes and 671 edges, with 191 miRNAs and 324 TF genes interacting with the hub genes (Fig. 7).

### Screening potential drugs

Following a rigorous screening process, we have successfully identified ten potential drug candidates that hold promise in the treatment of COVID-19 in conjunction with OSA. These drugs encompass acetaminophen (CTD 00005295), 5-Fluorouracil (CTD 00005987), genistein (CTD 00007324), estradiol (CTD 00005920), topotecan (CTD 00007293), POTASSIUM NITRATE (CTD 00001112), simvastatin (CTD 00007319), 7646–79-9 (CTD 00000928), Pinosylvin (CTD 00002139), and 2,4-dichlorophenoxyacetic acid (CTD 00007028). A comprehensive description of these drugs is provided in Table 3.

**Table 3:** Suggested top drug compounds for COVID-19 and OSA

Term	*P*-value	Adjusted *P*-value	Genes
acetaminophen CTD 00005295	5.67E-06	0.002117207	RB1;PCNA;CAV1;CD109;FTH1;UBE2T; NF1;SMARCAD1;PLS3;PLOD2;NCAPD2
5-Fluorouracil CTD 00005987	6.57E-06	0.002117207	RB1;PCNA;CAV1;PLK2;UBE2T;PLOD2;HIF1A
genistein CTD 00007324	7.70E-06	0.002117207	RB1;PCNA;CAV1;PLK2;UBE2T;NCAPD2;HIF1A
estradiol CTD 00005920	9.25E-06	0.002117207	RB1;PCNA;CAV1;PLK2;CD109;FTH1; UBE2T;NF1;PLS3;PLOD2;NCAPD2
topotecan CTD 00007293	2.16E-05	0.003806737	PCNA;FTH1;PLOD2;HIF1A
POTASSIUM NITRATE CTD 00001112	2.49E-05	0.003806737	PCNA;FTH1
simvastatin CTD 00007319	4.64E-05	0.004501554	RB1;PCNA;CAV1;HIF1A
7646–79-9 CTD 00000928	4.78E-05	0.004501554	PCNA;CAV1;PLK2;FTH1;UBE2T;PLS3; PLOD2;NCAPD2;HIF1A
Pinosylvin CTD 00002139	4.91E-05	0.004501554	RB1;PCNA;FTH1
2,4-dichlorophenoxyacetic acid CTD 00007028	5.43E-05	0.004501554	PCNA;FTH1

### Verification of the hub genes in clinical samples

**Fig. 8:**
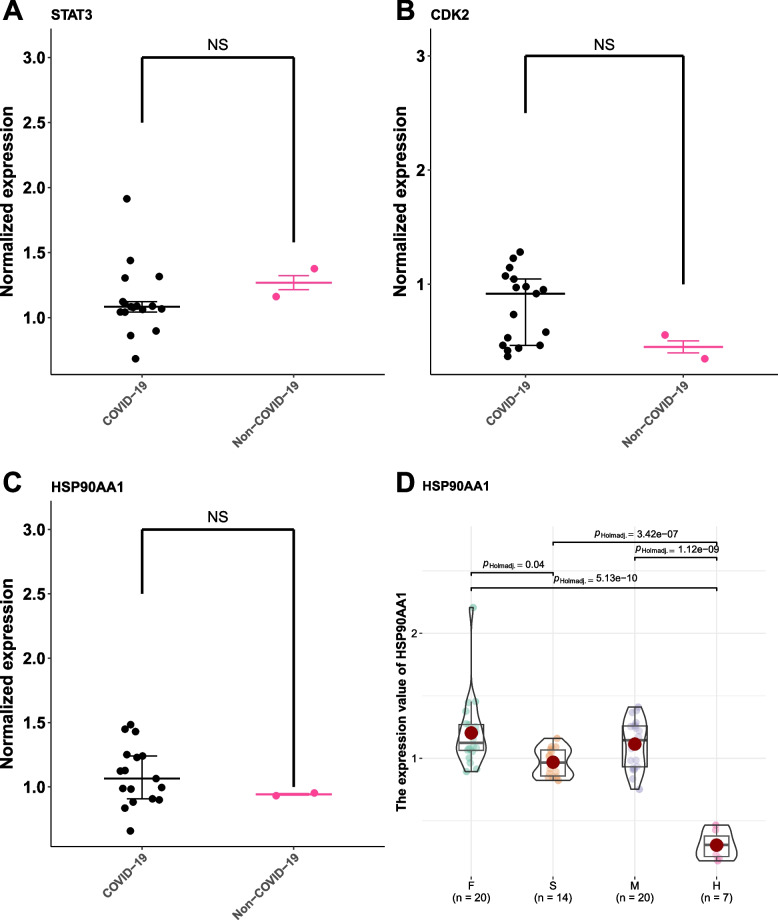
Validation of hub genes expression in COVID-19 lung tissue proteomics and plasma proteomics. **A** Validation of STAT3 expression in COVID-19 lung tissue proteomics. **B** Validation of CDK2 expression in COVID-19 lung tissue proteomics. **C** Validation of HSP90AA1 expression in COVID-19 lung tissue proteomics. **D** Plasma levels of HSP90AA1 protein from the serum samples of COVID-19. Data points are presented for each individual patient, shown as median values with interquartile ranges (F, *n* = 20; S, *n* = 14; M, *n* = 20; H, *n* = 7). The center line of each box indicates the median and the top and bottom of the box represent the 75th and 25th percentile values, respectively. NS, no significance

Among the central genes, we observed the discernment of corresponding protein entities solely for STAT3, CDK2, and HSP90AA1. Within the confines of COVID-19 pulmonary tissue specimens, the expression profiles of CDK2 and HSP90AA1 exhibited an elevation vis-à-vis control samples, while the expression profile of STAT3 demonstrated a reduction in relation to control samples, albeit without attaining statistical significance (Fig. 8A-C). Moreover, we embarked on an exploration of the prognostic implications encapsulated within the HSP90AA1 proteins present within clinical serum samples originating from diverse COVID-19 cases, each marked by disparate outcomes (Fatal (F) = 20, Severe (S) = 14, Mild (M) = 20, Healthy (H) = 7). Our findings unveiled a notable escalation in the plasma concentration of HSP90AA1 in the context of more severe manifestations of COVID-19 (Fig. 8D).

### Immune infiltration analysis and ROC curve

**Fig. 9:**
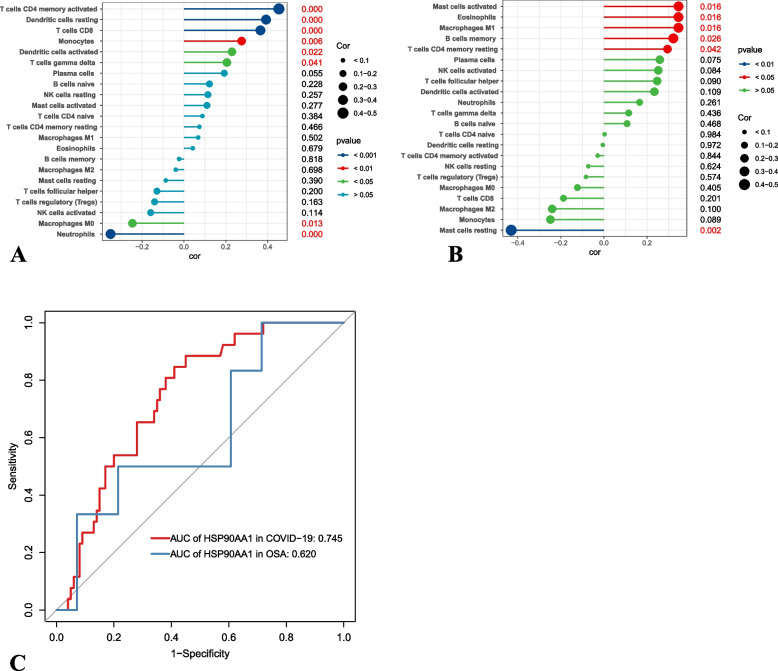
Relationship between HSP90AA1 and immune cell infiltration. **A** Relationship between HSP90AA1 and immune cell infiltration in COVID-19. **B** Relationship between HSP90AA1 and immune cell infiltration in OSA. **C** ROC analysis of HSP90AA1 protein in COVID and OSA

We undertook a comprehensive analysis of the intricate interrelationship existing between HSP90AA1 and immune cell populations, both in the context of COVID-19 and OSA. Within the ambit of COVID-19, a positive correlation was discerned between HSP90AA1 and activated CD4 memory T cells, as well as resting and activated dendritic cells, CD8 T cells, monocytes, and gamma delta T cells. Conversely, a negative correlation was observed between HSP90AA1 and neutrophils, along with M0 macrophages. In the landscape of OSA, HSP90AA1 exhibited positive correlations with resting CD4 memory T cells, memory B cells, M1 macrophages, eosinophils, and activated mast cells. On the other hand, a negative correlation was noted between HSP90AA1 and resting mast cells (Fig. 9A-B). The diagnostic potential of the HSP90AA1 gene was meticulously assessed through the construction of ROC curves. Within the confines of the COVID-19 dataset, the HSP90AA1 gene demonstrated commendable diagnostic efficacy (AUC: 0.745) in discriminating individuals afflicted by SARS-CoV-2 from their healthy counterparts. In the expanse of the OSA dataset, the HSP90AA1 gene achieved an AUC value of 0.620 (Fig. 9C).

## Discussion

Various observational studies have indicated that individuals afflicted with OSA exhibit a heightened vulnerability to severe SARS-CoV-2 infection [10, 15–17], albeit this association diminishes upon adjustment for other comorbidities (such as obesity and cardiovascular disorders) [33]. A Mendelian randomization study also yielded inconclusive evidence regarding the causal relationship between OSA and COVID-19. Conversely, a population-based study demonstrated that even after accounting for the effects of other comorbidities (e.g., obesity), OSA remains capable of doubling the risk of COVID-19-related adverse events, including hospitalization and death. Consequently, further comprehensive investigations are imperative to elucidate the intrinsic relationship between OSA and COVID-19. Within this study, fourteen feature genes shared by COVID-19 and OSA have been identified. Subsequently, through the construction of a protein–protein interaction network incorporating these common feature genes, we have identified 10 central genes (TP53, CCND1, MDM2, RB1, HIF1A, EP300, STAT3, CDK2, HSP90AA1, and PPARG). The subsequent analysis proceeded with functional enrichment analysis, TF-gene interactions, TF-miRNA interaction networks, and drug candidate screening.

Protein–protein interaction is the process by which two or more protein molecules form a protein complex through non-covalent bonds. At the molecular level, viruses invade cells and use a complex network of protein–protein interactions to manipulate cellular processes, and host cells initiate activation of innate antiviral defenses and adaptive immune systems to control viral replication through complex protein–protein interactions. Therefore, knowledge of protein–protein interactions is essential for understanding the virus-host relationship. Zhang et al. identified relevant genes in the virus-human protein interaction network, including UBL4A, GNB1, UMPS, POTEF, UBL4B, UBBP4, PARK2, PTPN11, and SCOC based on a random walk model [34]. In particular, Yang et al. developed the Human-Virus Interaction DataBase (HVIDB, http://zzdlab.com/hvidb/), which can accurately predict interactions between human host and viral proteins [35].

Currently, integrated machine learning based on multiple feature selection algorithms has been widely used to find biomarkers for diseases, which can screen potential biomarkers with high sensitivity, accuracy, and stability, and construct efficient and stable diagnostic models. Since the outbreak of COVID-19, numerous studies have analyzed transcriptomic, proteomic, metabolomic, and epigenomic data from COVID-19 patients based on machine learning to find reliable and stable COVID-19 biomarkers. Guo et al. developed a random forest machine learning model based on proteomic and metabolomic data from COVID-19 patients, which incorporated genes such as SAA2,ALB,CRP,SAA1,HABP2, and HP, and was able to discriminate well between non-severe and severe COVID-19 patients (AUC = 0.957) [36]. Using LASSO and ridge regression penalties, Zhou et al. identified a biomarker combination containing ORM1/AGP1, ORM2, FETUB, and CETP that demonstrated excellent reliability in distinguishing COVID-19 patients from healthy volunteers [31]. Cai et al. identified five biomarkers of COVID-19 by applying Boruta and minimum redundancy maximum relevance methods, which were PSMB8, COLCA2, FAM83A, LGALS3BP and IRF9. In addition, Cai et al. analyzed the methylation dataset of COVID-19 by utilizing the Monte Carlo feature selection method analyzed the methylation dataset of COVID-19 and found that EPSTI1, NACAP1, SHROOM3, C19ORF35 and MX1 as key features could significantly distinguish COVID-19 patients from non-COVID-19 patients [37, 38].

Prior investigations have identified five of these central genes (TP53, HIF1A, STAT3, HSP90AA1, PPARG) to be linked to the pathogenesis of both COVID-19 and OSA. The tumor suppressor gene TP53, a crucial participant in the apoptotic signaling cascade, assumes a significant role in monitoring cellular division and possesses reparative effects on aberrant division and cellular damage. Multiple studies have revealed a downregulation of TP53 gene expression in patients infected with SARS-CoV-2, consequently fostering viral replication and increasing the likelihood of tumorigenesis [39–42]. Intriguingly, periodic intermittent hypoxia has been implicated as a causative factor for non-small cell lung cancer in TP53fl/fl mice, thereby facilitating accelerated tumor growth [43]. Hence, we postulate that individuals afflicted with the concomitant presence of these two ailments are predisposed to a heightened risk of cancer or tumor development, ultimately resulting in diminished overall survival rates.

HIF1A (hypoxia-inducible factor 1-alpha), serves as a prominent regulator of cellular and systemic homeostatic responses to hypoxia by orchestrating the transcriptional activation of numerous genes. These genes encompass those involved in energy metabolism, angiogenesis, apoptosis, and others whose protein products enhance oxygen delivery or facilitate metabolic adaptation to hypoxic conditions [44]. Notably, previous investigations have indicated that the prognosis of COVID-19 is significantly influenced by the cytokine storm triggered by SARS-CoV-2. Transcriptome analyses have unveiled that SARS-CoV-2 infection induces mitochondrial damage and the generation of mitochondrial-derived reactive oxygen species (Mito-ROS), thereby promoting the expression of HIF-1A and consequent SARS-CoV-2 infection and cytokine production [45, 46]. Furthermore, HIF-1A assumes a vital role in regulating metabolic pathways and inflammatory processes [47]. The dysregulation of the HIF-1A pathway contributes to the development of various diseases, including cancer, cardiovascular disease, and Alzheimer's disease [48–51]. Importantly, many of these disorders have been demonstrated to be associated with severe SARS-CoV-2 infection, thereby suggesting the prognostic impact of HIF-1A on COVID-19 [52]. On the other hand, numerous studies have illustrated the close association between HIF-1A and OSA, given that OSA is characterized by chronic intermittent hypoxia (CIH). CIH stimulates the upregulation of the hypoxia-inducible transcription factor HIF1A, contributing to the independent risk of OSA for atherosclerosis and heightened cardiovascular mortality. Mechanistically, hypertension, dyslipidemia, insulin resistance, systemic inflammation, and oxidative stress are among the underlying mechanisms through which CIH accelerates atherosclerotic disease [53]. A recent study has demonstrated that mice exposed to CIH exhibited impaired clearance of triglyceride-rich lipoproteins. Subsequent investigations have suggested that this phenomenon may be attributed to the transcriptional regulation of HIF-1A, which upregulates the expression of the lipoprotein lipase (LPL) inhibitor (Angptl4), thereby inactivating LPL and impeding lipid clearance [54]. Additionally, CIH exacerbates non-alcoholic fatty liver disease (NAFLD) by inducing hepatic oxidative stress via the activation of the HIF-1A gene [55]. Furthermore, HIF-1 has been implicated in the pathological processes of hypoxia and has been associated with various other diseases such as diabetes mellitus, idiopathic pulmonary fibrosis, pulmonary arterial hypertension, systemic hypertension, myocardial injury, and cognitive deficits [56–61]. In summary, the myriad metabolic and pathophysiological responses elicited by intermittent hypoxia in individuals with OSA may contribute to their susceptibility to COVID-19 and portend a poorer prognosis.

Inflammation constitutes a hallmark feature of both OSA and SARS-CoV-2 infections. Hypotheses suggest that alterations in the gut microbiota, which are associated with COVID-19 and OSA, may compromise the integrity of the intestinal barrier, thereby facilitating the translocation of bacteria into the systemic circulation and subsequently promoting systemic inflammation [8]. Notably, systemic inflammation is particularly pronounced in the context of COVID-19. STATs (signal transducer and activator of transcription) are well-known components of the JAK/STAT signaling pathway. Previous investigations have revealed that the viral component of SARS-CoV-2 induces malfunctioning of STAT1 and compensatory overactivation of STAT3. Consequently, a positive feedback loop between STAT3 and plasminogen activator inhibitor-1 (PAI-1) is established in SARS-CoV-2-infected cells, thereby perpetuating a cycle of activation that triggers the release of pro-inflammatory cytokines and chemokines [62].

In a transcriptomic analysis, the connection between HSP90A family class A member 1 (HSP90AA1) and the quantity of SARS-CoV-2 RNA was unveiled [63]. Moreover, HSP90AA1 was identified as a risk factor for the coexistence of COVID-19 and cardiovascular disease [64]. Notably, HSP90 levels were significantly elevated in patients with OSAS when compared to control subjects [65]. These findings suggest that HSP90 may potentially play a role in the progression of both of these diseases.

PPARG (peroxisome proliferator-activated receptor gamma), a crucial transcription factor, governs both inflammation and fatty acid metabolism. Studies have demonstrated that PPARG agonists can enhance the expression of ACE2, thereby contributing to the invasion of SARS-CoV-2 [66]. Furthermore, OSA is characterized by metabolic dysfunction and obesity, and the PPARG gene is implicated in the regulation of lipid metabolism and glucose homeostasis. This suggests that PPARG may also play a potential role in the pathophysiological mechanisms underlying the comorbidity of COVID-19 and OSA [67]. As for the other five central genes (CCND1, MDM2, RB1, EP300, CDK2) in the study, their associations with OSA patients have not been reported. Therefore, future investigations could delve into exploring the potential mechanisms that underlie the comorbidity between COVID-19 and OSA from additional perspectives.

Condensed chromosomes, centromeric regions, autolysosomes, and molecular functions related to DNA insertion or deletion was enriched in this study. The primary constriction along a condensed chromosome is known as the centromere region, and the end of the chromosome as the telomere. These regions are essential for the proper integrity and segregation of chromosomes, as well as for the hierarchy of order within the cell nucleus. A study conducted at the Federal University of São Paulo in Brazil shows that the telomere shortening that naturally occurs with aging and is accelerated by OSA can be mitigated by the use of continuous positive airway pressure, indicating that accelerated telomere shortening due to OSA can therefore lead to premature cell aging [68]. It is now well established that SARS-CoV-2 can enter host cells via the ACE2 receptor. Interestingly, the gene encoding ACE2 is located on an X chromosome, and according to Lyon's theory, one of the two X chromosomes is transcriptionally silenced, a process that results in the condensation of an X chromosome into a compact structure known as a barr body [69, 70]. Autolysosome is formed when an autophagosome or phagosome fuses with a lysosome [71]. As a marker of autolysosomal degradation, P62 protein expression in blood cytotoxic T cells was increased in OSA patients compared to primary snoring patients, indicating that OSA patients had impaired autophagy activity [72]. In COVID-19, the SARS-CoV-2 protein ORF3a affects autolysosome formation by hindering the assembly of the SNARE complex, and furthermore, SARS-CoV-2 infection-induced autolysosome accumulation facilitates progeny virus propagation [73–75]. As for DNA insertion or deletion, it was shown that angiotensin-converting enzyme gene insertion/deletion polymorphisms differed significantly in OSA disease groups with different severities and may be involved in the pathogenesis of hypertension [76, 77]. In COVID-19, insertion-and-deletion mutations in several genes including Nsp2, Nsp3, S1, and ORF8 genes have been observed, which may provide further insights into the developmental process of SARS-CoV-2 [78, 79].

The study revealed that gas homeostasis may be a crucial shared pathogenic mechanism in COVID-19 and OSA, as indicated by the analysis of GO enrichment. Gas homeostasis refers to the tightly regulated maintenance of oxygen and carbon dioxide levels in the human body, involving multiple pathways. Recent research has suggested the involvement of these pathways in the pathogenesis of both COVID-19 and OSA. Hypoxia-inducible factors (HIFs) are known regulators of cellular responses to low oxygen levels. We have discussed in detail the significant role of HIF1A in the development of COVID-19 and OSA patients. Furthermore, OSA patients experience chronic intermittent hypoxia, while the increased production of pro-inflammatory cytokines in COVID-19 also affects gas exchange and disrupts gas homeostasis [80]. Oxidoreductase activity refers to the enzymatic activity involved in redox reactions, which is closely associated with oxidative stress [81]. It is increasingly recognized that COVID-19 pathogenesis and immunopathogenesis are closely linked to oxidative stress and cytokine storm. Oxidative stress contributes to the development of endothelial dysfunction, while the cytokine storm exacerbates this effect. Ultimately, these events trigger the activation of the blood clotting cascade, leading to blood coagulation and microvascular thrombosis. In the case of OSA, intermittent hypoxia and reoxygenation during sleep-disordered breathing also induce oxidative stress and inflammation[82, 83]. This resulting oxidative damage further contributes to the development and worsening of complications associated with OSA, including obesity, metabolic dysfunction, cardiovascular diseases, and cognitive impairment [82, 84, 85]. DNA repair and replication pathways play vital roles in maintaining the stability of the genome and ensuring cell survival. Recent studies have revealed that these pathways may also be involved in the pathogenesis of COVID-19 and OSA. Emerging evidence suggests that viral infection can induce DNA damage and trigger an altered DNA damage response. Specifically, certain products of SARS-CoV-2, such as ORF6 and NSP13 proteins, disrupt the DNA damage response kinase CHK1 and reduce the production of deoxynucleoside triphosphate (dNTP), which consequently leads to DNA damage response. In the case of OSA, increased levels of reactive oxygen species and repeated hypoxic conditions also disrupt DNA repair mechanisms and contribute to enhanced DNA damage [86]. In summary, gas homeostasis, oxidoreductase activity, and DNA repair and replication pathways may contribute to the shared pathogenesis of COVID-19 and OSA. Further studies are required to fully understand the precise roles of these pathways in these diseases and to explore their potential as therapeutic targets.

In this study, ten drug candidates were identified. To further strengthen the credibility of the results, we searched databases of information about drugs for the treatment of COVID-19, including DockCoV2 and DrugBank, assembled by Huang et al. [87–90]. Of these, Topotecan and Estradiol had docking scores of -16.8 and -10.7, respectively, indicating potential as therapeutic agents.

It is important to acknowledge the limitations of this study. Firstly, the analysis was conducted using data from patients diagnosed with COVID-19 and OSA, rather than individuals specifically diagnosed with both disorders. This may impact the generalizability of the findings to the co-occurrence of COVID-19 and OSA. Additionally, it should be noted that the identified genes were not experimentally validated in OSA samples.

## Conclusions

In conclusion, this investigation revealed a set of genes, molecular networks, and signaling pathways that are associated with both COVID-19 and OSA. These findings provide insights and potential avenues for further research into the molecular mechanisms underlying these two diseases. The study highlights a shared regulatory scheme and signaling mechanism between COVID-19 and OSA.

### Supplementary Information

**Supplementary 1.**

## Data Availability

The datasets (accession number: GSE157103 and GSE75097) generated and analysed during the current study are available in GEO (https://www.ncbi.nlm.nih.gov/geo), which are public functional genomics data repositories. In addition, we uploaded the data and scripts to Zenodo (https://zenodo.org/records/10591549).
