# Supplementary material for: Identification of biomarkers and pathways for the SARS-CoV-2 infections in obstructive sleep apnea patients based on machine learning and proteomic analysis
Source: BMC Pulm Med. 2024 Mar 5;24:112. doi: 10.1186/s12890-024-02921-1 (PMC10913609; doi:10.1186/s12890-024-02921-1)
Supplement: Supplementary file 1 — Supplementary 1. [file 12890_2024_2921_MOESM1_ESM.pdf]

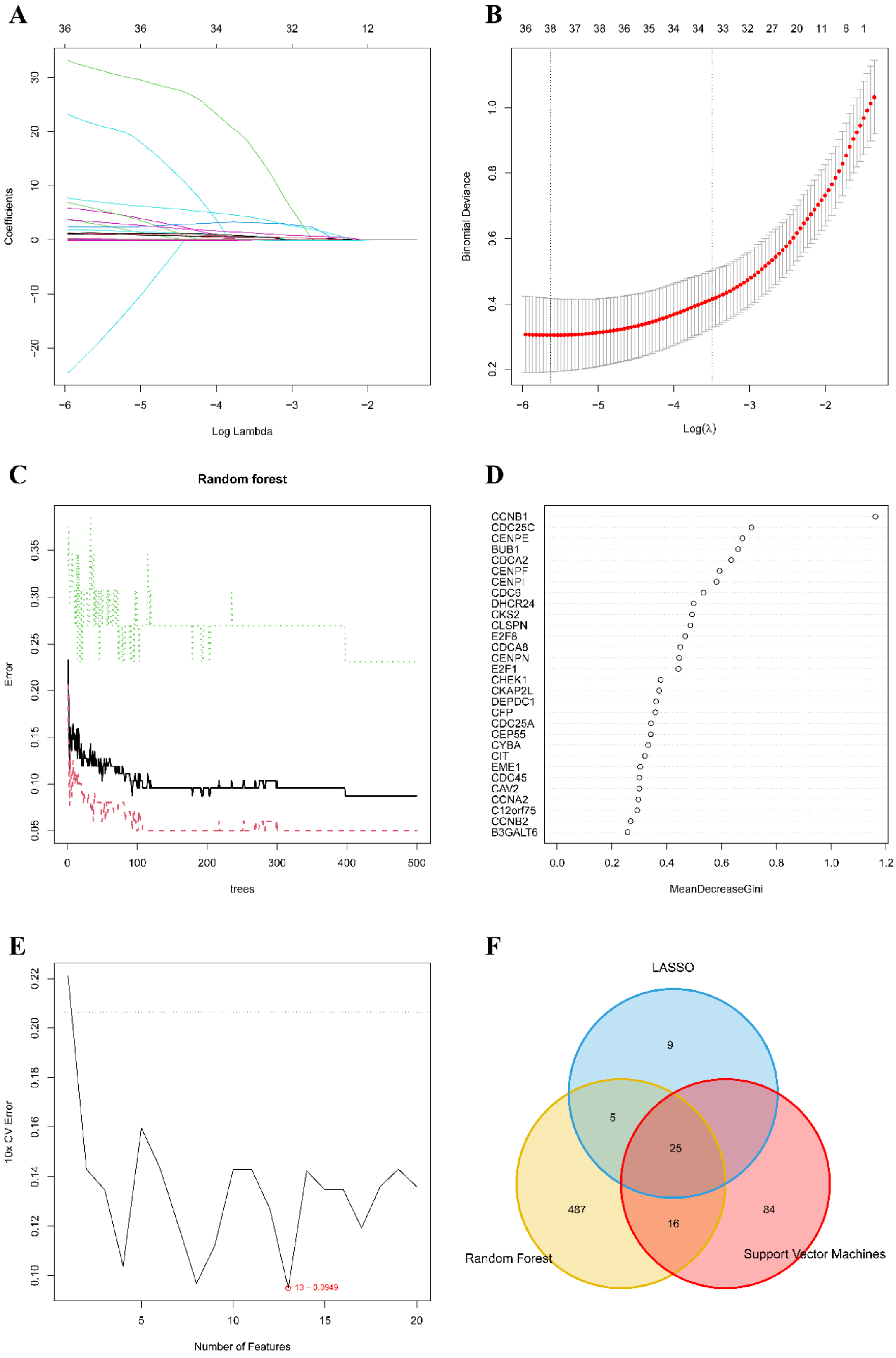

Figure S1 Screening of feature genes via multiple machine-learning algorithms in COVID-19. (A-B) Screening of feature genes via LASSO regression. (A) Different colors represent different genes. (B) LASSO for eight candidate genes and the error bars mean the fluctuation range of Binomial Deviance. (C-D) Screening of feature

genes via random forest algorithm. (C) Relationship between the overall error rate and the number of trees. The red, black, and green lines represent the COVID-19, overall and control groups respectively. (D) The variables are ranked in order of importance. (E) Screening of feature genes via support vector machines algorithm. (F)Intersection of feature genes via three machine-learning algorithms.

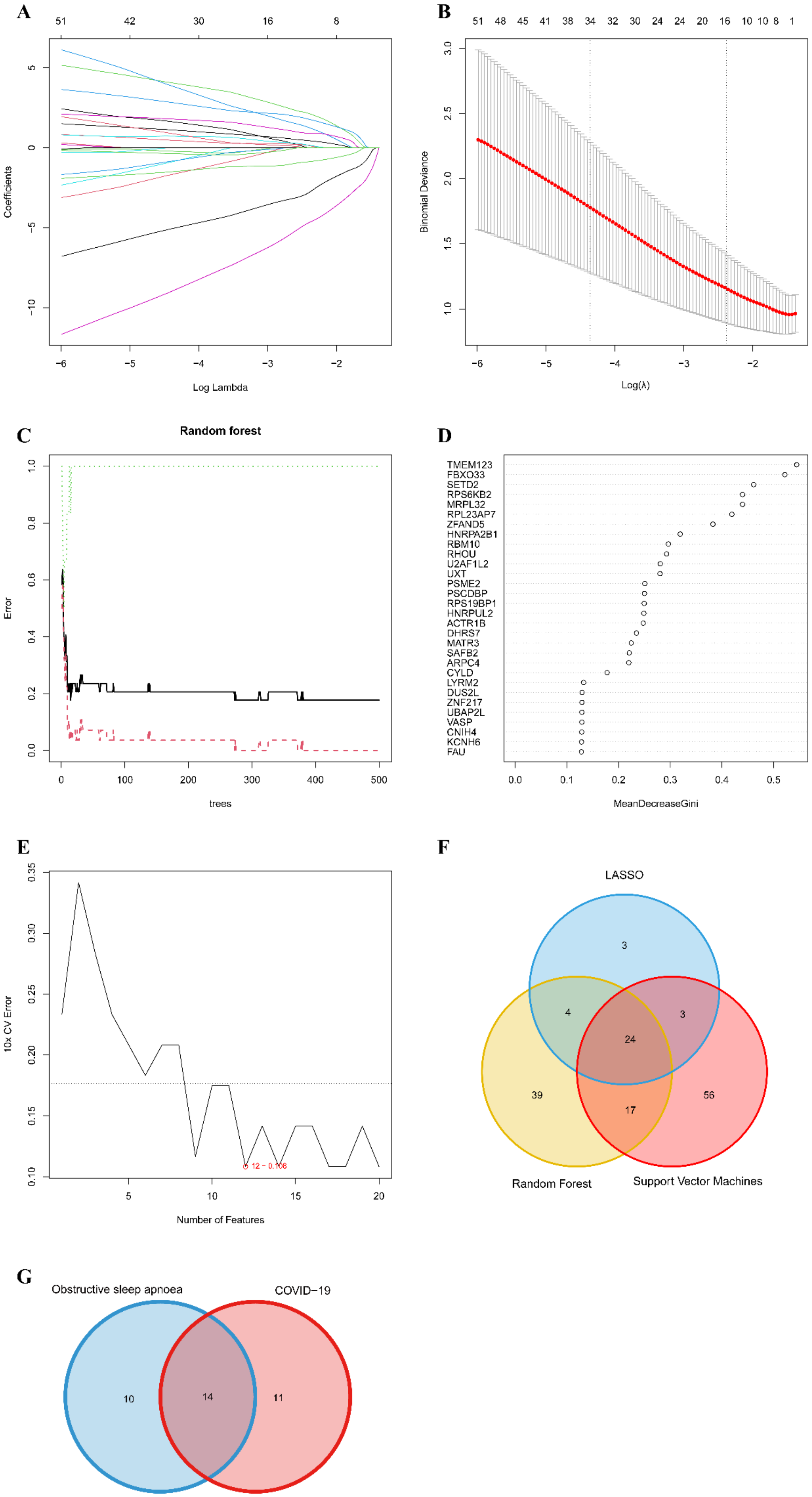
A
51 42 30 16 8
Coefficients
Log Lambda
B
51 48 45 41 38 34 32 30 24 24 20 16 10 10 8 1
Binomial Deviance
Log(λ)
C
Random forest
Error
trees
D
TMEM123
FBXO33
SETD2
RPS6KB2
MRPL32
RPL23AP7
ZFAND5
HNRPA2B1
RBM10
RHOU
U2AF1L2
UXT
PSME2
PSCDBP
RPS19BP1
HNRPUL2
ACTR1B
DHRS7
MATR3
SAFB2
ARPC4
CYLD
LYRM2
DUS2L
ZNF217
UBAP2L
VASP
CNIH4
KCNH6
FAU
MeanDecreaseGini
E
10x CV Error
12 - 0.108
Number of Features
F
LASSO
3
4
3
24
39
17
56
Random Forest
Support Vector Machines
G
Obstructive sleep apnoea
COVID−19
10
14
11

Figure S2 Screening of feature genes via multiple machine-learning algorithms in OSA. (A-B) Screening of feature genes via LASSO regression. (A) Different colors represent different genes. (B) LASSO for eight candidate genes and the error bars mean the fluctuation range of Binomial Deviance. (C-D) Screening of feature genes via random forest algorithm. (C) Relationship between the overall error rate and the number of trees. The red, black, and green lines represent the OSA, overall and control groups respectively. (D) The variables are ranked in order of importance. (E) Screening of feature genes via support vector machines algorithm. (F)Intersection of feature genes via three machine-learning algorithms. (G) Common feature genes representation through a Venn diagram. The two datasets showed an overlap of fourteen feature genes.
